# Interfacial Charge Transfer Regulates Photoredox Catalysis

**DOI:** 10.1021/acscentsci.3c01561

**Published:** 2024-02-26

**Authors:** Chen Ye, De-Shan Zhang, Bin Chen, Chen-Ho Tung, Li-Zhu Wu

**Affiliations:** † Key Laboratory of Photochemical Conversion and Optoelectronic Materials, New Cornerstone Laboratory, Technical Institute of Physics and Chemistry, The Chinese Academy of Sciences, Beijing 100190, P. R. China; ‡ School of Future Technology, University of Chinese Academy of Sciences, Beijing 100049, P. R. China

## Abstract

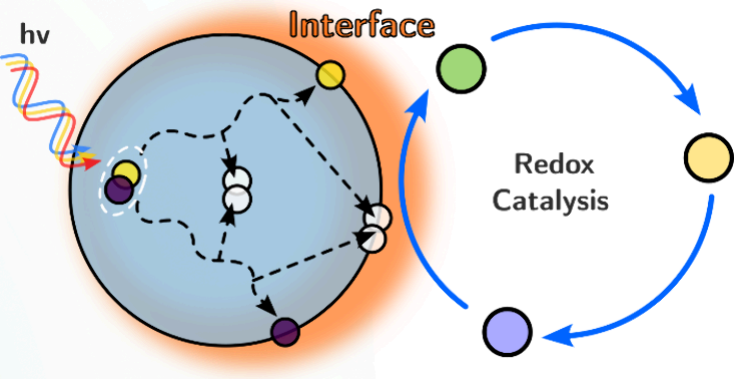

Photoredox catalytic processes offer the potential for precise chemical reactions using light and materials. The central determinant is identified as interfacial charge transfer, which simultaneously engenders distinctive behavior in the overall reaction. An in-depth elucidation of the main mechanism and highlighting of the complexity of interfacial charge transfer can occur through both diffusive and direct transfer models, revealing its potential for sophisticated design in complex transformations. The fundamental photophysics uncover these comprehensive applications and offer a clue for future development. This research contributes to the growing body of knowledge on interfacial charge transfer in photoredox catalysis and sets the stage for further exploration of this fascinating area of research.

## Abstract 2

This study delves into interfacial photoredox catalysis with QDs, emphasizing interfacial charge transfer’s role in precise chemical reactions and highlighting its potential for complex transformations, thus advancing knowledge in the field.

## 1 Introduction

**Figure 1:**
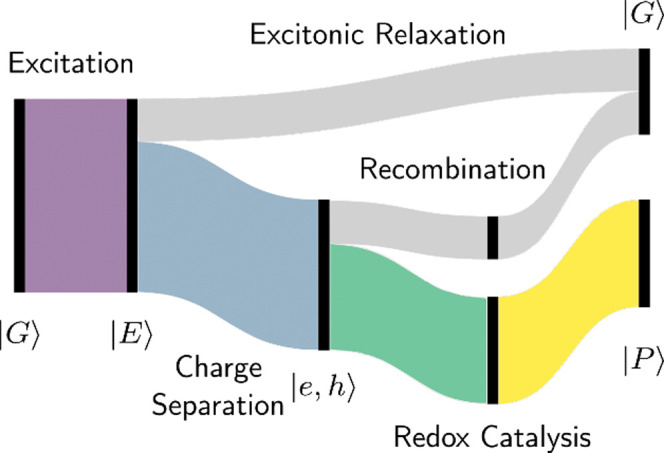
Flow diagram of the mechanism of photoredox catalysis.

Photoredox catalysis is a robust and versatile strategy that employs light to initiate chemical reactions, which excels in overcoming the limitations associated with conventional catalysis.^1^ The photocatalytic approach facilitates diverse transformations, including water splitting, carbon dioxide reduction, and organic synthesis to obtain useful chemicals and materials. The fundamental steps of photoredox catalysis can be succinctly summarized as a journey of photon flux (Figure 1). Light-harvesting materials absorb photons and generate charges. The photon-generated charges undergo separation and finally migrate to the reactive centers to achieve the target redox reactions. Charge separation is a crucial step that enables redox reactions to occur, while charge recombination acts as a competing channel for the target transformations, and the overall efficiency of the system depends on the balance between productive charge separation and redox reactions and competing charge recombination.

Over the past decades, in-depth investigations into the mechanisms of molecular photoredox catalysis have established a standard tool for synthetic chemists. However, the photoredox mechanism at a heterogeneous photocatalyst’s interfaces is more complicated than that in homogeneous phases. The complexity arises from the anisotropic effects and spatial distribution of physical properties, resulting in highly intricate kinetics, particularly at the interface, within heterogeneous systems. Merely applying the classic electron transfer theory, primarily formulated for homogeneous systems, fails to effectively address numerous practical challenges encountered under heterogeneous conditions. The comprehensive understanding of charge kinetics in practical and complex systems, particularly at the surfaces of heterogeneous photocatalysts, needs reevaluation and further investigation.^2^

The preceding discussion emphasizes the complexity and diversity of interfacial charge transfer, which presents both challenges and opportunities in the pursuit of the ultimate goal of photoredox catalysis.^3,4^ Extensive research has significantly contributed to the advancement of photocatalysis in the solution phase by exploring the fundamental steps of homogeneous photoredox catalysis. The interface in the hybird system not only offers advantages in charge kinetics but also promotes photoredox catalysis from a catalytic perspective. The interface offers an opportunity for rational preassembly and optimized geometry or interaction, favoring the desired reaction. The interfacial region assumes a pivotal role in determining the efficiency and selectivity of photoredox reactions, demanding a comprehensive theoretical framework. In this contribution, we aim to emphasize the key points of photoredox catalysis kinetics and highlight future opportunities for regulating photoredox catalysis at interfaces.

## 2 Kinetic Process at the Interface

**Figure 2:**
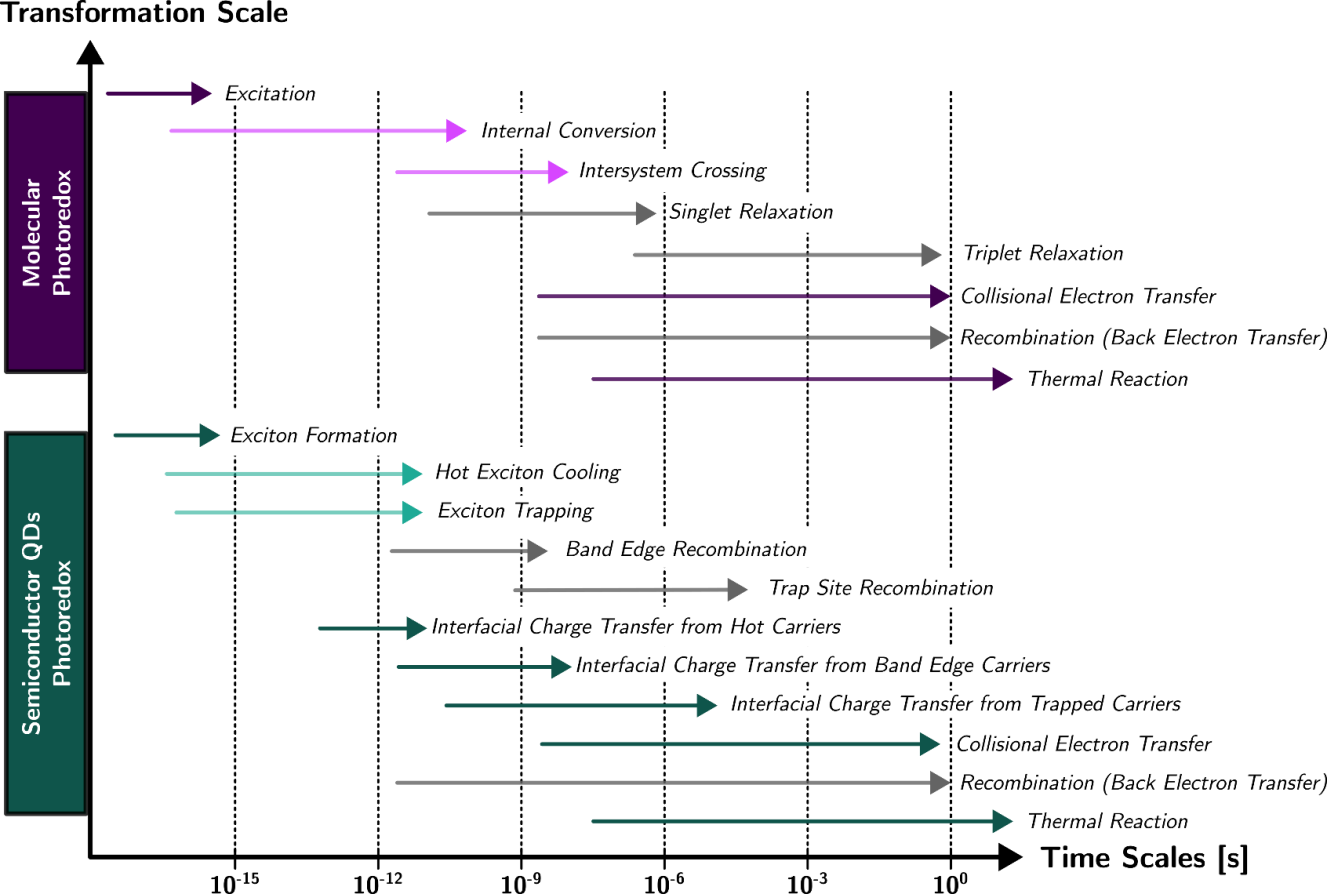
Schematic illustration of the time scale of each single step in photoredox catalysis through diffusion and through interfacial interaction.

Photoredox catalysis can be broadly classified into two categories: exciton evolution coupled with solution conversion. Excitons can be generated from interactions between light and matter in less than a femtosecond, while the final thermal reactions take milliseconds to seconds to occur (Figure 2).^5^ The primary mechanism of photoredox catalysis can be understood as a game theory model that encompasses various channels and rates. Charge separation and recombination follow the fundamental principles of photoinduced electron transfer, which can be quantitatively understood using Marcus theory. This theoretical framework establishes a relationship between the rates of these processes and several key factors, including the free energy difference between the reactants and products, the reorganization energy, and the electronic coupling between the donor and acceptor molecules (eq 1).^6^

The rate constant *k*_*ct*_ is a first-order rate constant that describes the intrinsic rate of charge transfer during a contact interaction.
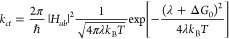
1where |*H*_ab_|^2^ is the electronic coupling strength between the charge donor and charge acceptor, λ is the reorganization energy, and Δ*G*_0_ is the energetic driving force.

In homogeneous solution systems, excited states are found in close proximity to the sensitizer species and charge separation occurs through diffusive collisions. The reactants move toward the photosensitizers and subsequently become activated through photoinduced electron transfer (eq 2).
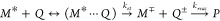
2

The significant difference in time scales poses a challenge for the overall reactions.^7^ Therefore, it is crucial to regulate the alignment of energy and rates to achieve highly efficient photoredox catalysis.^8,9^ The triplet photocatalysts are commonly employed in molecular photoredox catalysis, while the utilization of singlet chromophores is limited.

The detailed kinetics of diffusive charge transfer can be described by either the Smoluchowski or Collins–Kimball theory, each based on different assumptions. The most common cases at low viscosity and steady state can be approximated using the simplified form (eq 3), which was first proposed by Marian Smoluchowski:
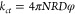
3where *R* is the reactive distance of close approach, *D* is the mutual diffusion coefficient, *N* is Avogadro constant with adjusted dimensions, and φ is the efficiency of electron transfer at contact. For diffusion-controlled electron transfer, the efficiency φ is 1. The rate constant is a second-order rate constant with an upper limit typically around 10^10^ M^–1^ s^–1^ in nonviscous environments. Recombination in solution can be considered as the unsymmetric reverse reaction of photoinduced charge separation, leading to relaxation to the neutral ground state through back charge transfer.

The photoinduced charge separation process on the interface is more complex than in solution due to the introduction of extra energy levels and potential reaction pathways, which profoundly impact the kinetics and efficiency. The interface can be constructed in different heterogeneous systems, including nanocluster systems, covalent frameworks, photoelectrodes, supramolecular structures, and so on. The various types of hybrid systems tremendously increase the diversity and complexity of the interfacial process. However, the fundamental principle of how photogenerated charges are transferred and utilized in redox catalysis can be well elucidated in the carefully selected model systems. Among the promising heterogeneous photocatalysts, semiconductor quantum dots (QDs) exhibit extraordinary advantages. They possess high light-harvesting efficiency, surface tunability, and compatibility, making them indispensable in the design, optimization, and industrial application of modern organic synthesis within the field of organic chemistry.^10−13^ QDs cannot be treated as independent entities. For example, QDs achieve charge separation via the immediate dissociation of excitons. Photogenerated charges can undergo additional spatial separation by interacting with reactants. The kinetics of photoinduced electron transfer on a surface can be first-order or second-order, depending on surface bonding and reactant diffusion. The preceding discussion emphasizes the complexity and diversity of interfacial charge transfer in QD–molecule systems, which present both challenges and opportunities in the pursuit of the ultimate goal of photocatalytic redox transformations.^3,4^

**Figure 3:**
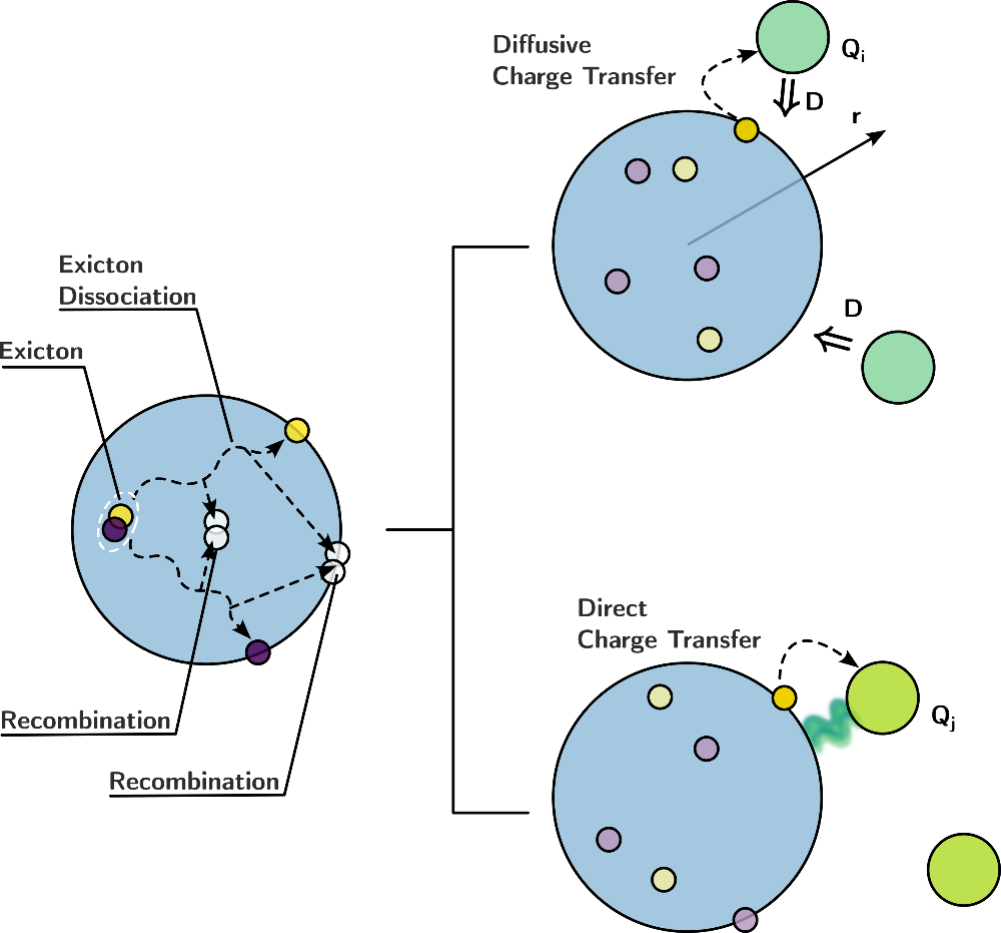
Schematic illustration of the charge kinetics of QDs and the photoinduced electron transfer at the QD surface.

When reactants diffuse toward one another without adhering to the surface, the kinetics might display second-order behavior, in line with the diffusive reaction theory (Figure 3, Diffusive Charge Transfer). In such cases, the rate constant is influenced by the diffusion coefficient and the distance between the reactants. The charge transfer kinetics on QD surfaces mirror behaviors observed in the solution phase. Yet, the surfaces of QDs possess dangling bonds and defect sites, facilitating the chemical attachment of molecular reactants. These attached molecular reactants can function as surface ligands, bolstering the structural stability and suspension attributes of QDs.^14^ When reactants are affixed to the surface, charge transfer can proceed via a direct surface reaction, leading to first-order kinetics (Figure 3, Direct Charge Transfer). Weiss et al. illustrated both reaction mechanisms within a single sample.^15^ During photoinduced electron transfer from PbS QDs to 1,4-benzoquinone (BQ), two distinct components emerged: a swift electron transfer on the picosecond time scale associated with the direct transfer from QDs to the chemically attached BQ ligands and a more prolonged component involving diffusion-controlled charge transfer.

Pioneering studies conducted by Lian et al. have demonstrated that efficient electron transfer from the band gap state of QDs at the interfaces of CdS QDs/Rhodamine B and CdSe QDs/Rebipyridyl complexes can occur at an astonishingly rapid rate of 1–10 ps.^16^ This process even outpaces exciton–exciton annihilation, which typically takes 10–100 ps.^17^ These findings underscore the potential of photoinduced direct charge transfer as a highly efficient process for catalytic redox reactions. Sophisticated designs enable photoinduced electron/hole transfer at the interface between QDs and molecules to occur on a subpicosecond time scale.^18−20^ This mechanism has been successfully employed in the assembly of QDs with molecular catalysts, including Rebipyridyl complexes and [FeFe]-hydrogenase model complexes, among others.^21−23^ The ultrafast interfacial charge transfer is also observed in QDs combined with metals or metal oxides.^24−26^ Charged metals and metal oxides can further serve as cocatalysts to drive thermal redox catalysis, presenting exciting prospects for highly efficient photocatalytic redox reactions.

**Figure 4:**
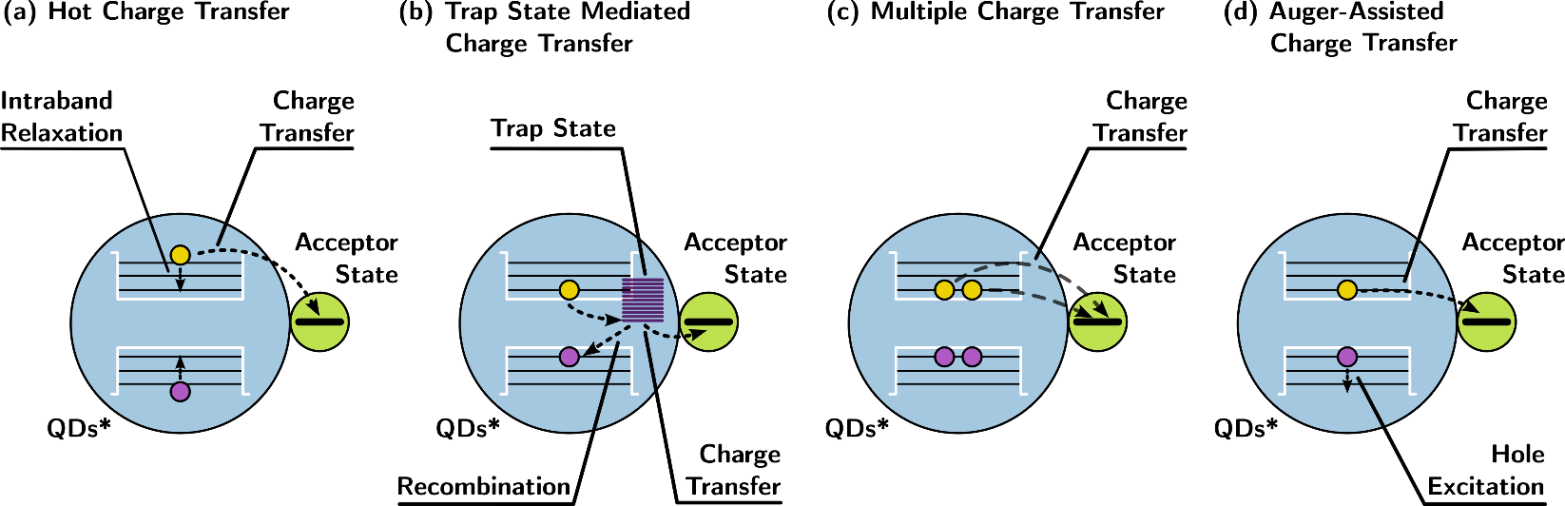
Schematic illustration of the special types of interfacial charge transfer from QDs to surface bound molecules. Electron transfer is selected here as the representative case for the charge transfer.

In contrast to molecular photocatalysts, which primarily utilize the lowest excited states as dictated by Kasha’s rule, heterojunction photocatalysts like QDs tend to involve higher excited states when interacting with redox reactants. The correlated and coupled energy states of QDs increase the complexity of the system while also expanding the range of potential reaction pathways.^27,28^ Hot excitons, located above the lowest bandgap excitons in QDs, can rapidly undergo charge transfer at the QD–molecule interface, facilitating the desired redox reactions.^29^ By appropriately designing the QD–molecule or QD–cocatalyst interface, charge transfer at a subpicosecond level can be achieved, surpassing the ultrafast intraband cooling process (Figure 4a).^30−33^ However, the presence of defect states in QDs significantly affects interfacial charge transfer.^34^ These defect sites can either trap photogenerated charges from the bandgap or hot excitons, acting as charge transfer relays^35,36^ or recombination centers,^37,38^ leading to opposing directions of interfacial charge separation (see Figure 4b). QDs exhibit highly degenerate and quasi-discrete energy levels within a single unit, making them susceptible to multiexciton processes (Figure 4c).^39^ This characteristic gives rise to nonlinear charge transfer at the QDs interface, as demonstrated by the research of Wasielewski and Co.^40^ By adjusting the excitation power, interfacial electron transfer from CdS QDs to surface-bound molecules can be tuned from linear one-electron transfer to nonlinear bi-electron transfer, resulting in significant differences in charge recombination rates of up to 4 orders of magnitude. Another notable process involved in interfacial charge transfer is the auger-assisted charge transfer. Auger-assisted electron transfer combines interfacial electron transfer with hole excitation to deeper positions in the valence band, thereby promoting electron transfer and the desired target reactions (Figure 4d).^41,42^

According to Marcus theory, the rate of charge transfer is heavily influenced by the electronic coupling (|*H*_*ab*_|^2^) between the donor QDs and the acceptor molecule. By manipulating and enhancing the wave functions of QD–molecular hybrid states, photoinduced charge transfer at the interface can be engineered.^43^ For molecules with a common core structure, the electron coupling is correlated with the distance between the charge donor and acceptor, as described by eq 4:
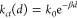
4where the factor β corresponds to the barrier of electron tunnelling.^44^ Adjusting the charge transfer distance at the QD–molecule interface can be achieved by various approaches. One feasible method is the construction of a core–shell structure for the QDs, where the thickness of the shell can be tuned accordingly.^45^ Another viable approach involves increasing the length of the anchor ligand, although careful consideration of the actual distance is necessary.^46^ Weiss et al. revealed that the addition of more alkyl groups may not alter the QD–chromophore distance, thereby keeping the interfacial charge transfer rate unchanged.^47^

**Figure 5:**
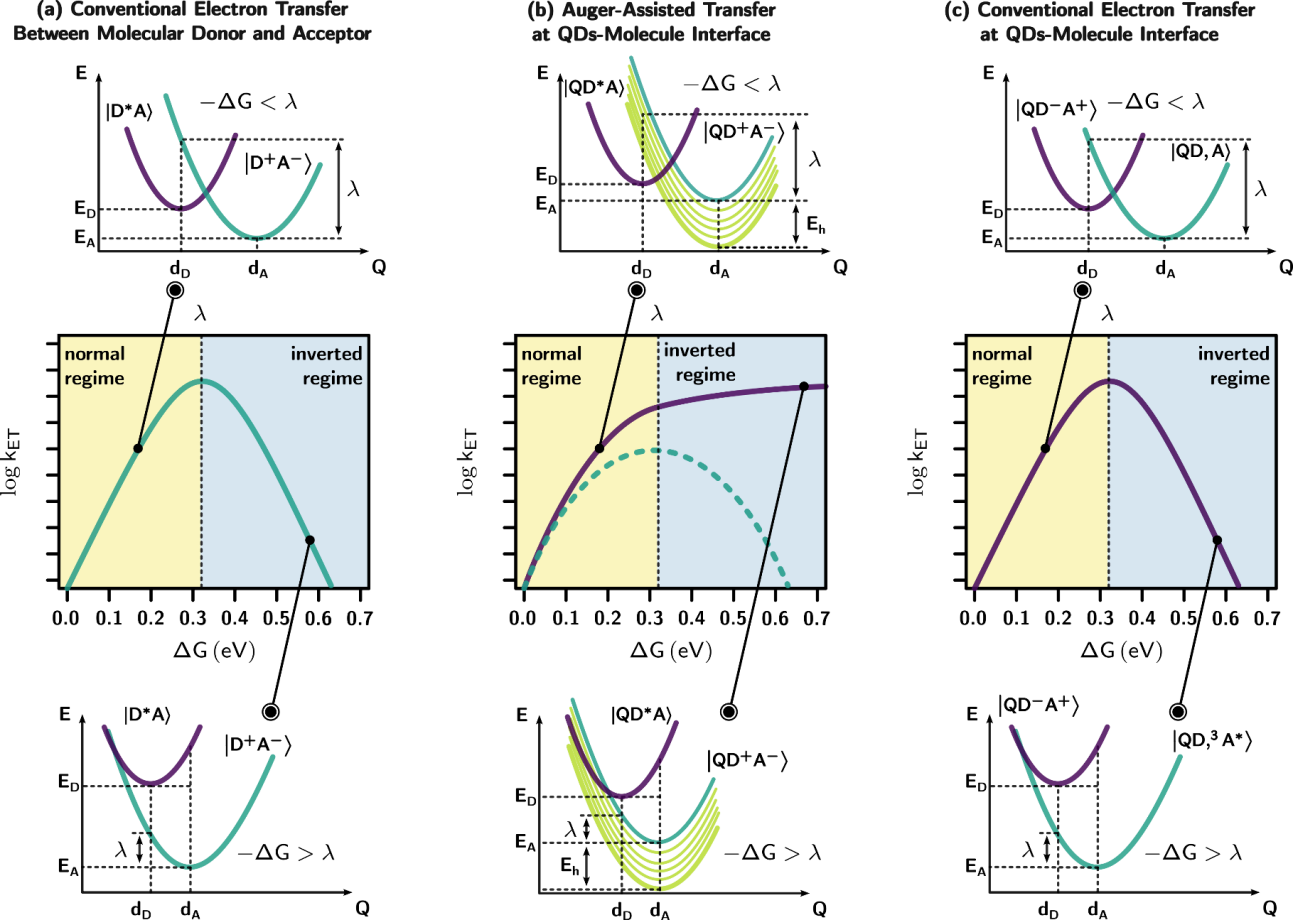
Schematic illustration of (a) conventional electron transfer with Marcus normal regime and Marcus inverted regime of molecular systems; (b) Auger-assisted electron transfer without Marcus inverted regime at the QD–molecule interface; (c) conventional electron transfer at the QD–molecule interface.

The energy dependence of the electron transfer rate follows a typical pattern observed in a Marcus-type electron transfer. As the free energy difference increases, the electron transfer rate initially rises and then declines due to the parabolic function in the power exponent (eq 1). The regime characterized by a continuously increasing rate is known as the “normal regime”, while the region with a decreasing rate is referred to as the “inverted regime” (Figure 5a). However, the Marcus inverted regime is generally absent at the QD–molecule interface, as noted by the work of Lian, due to the influence of auger-assisted charge transfer, which deviates from Marcus theory.^41^ In QDs, the dynamics of electrons and holes are strongly correlated due to quantum confinement, and the valence band distribution of QDs must be considered. The rate constant for auger-assisted charge transfer can be calculated using eq 5:
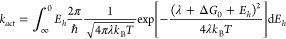
5where *E*_*h*_ is the hole excitation energy. The continuous intraband transition of holes can benefit auger-assisted electron transfer, resulting in the absence of the Marcus inverted regime (Figure 5b).^48^ Wu et al. indicate that the excitonic electron transfer occurring at the QD–molecule interface exhibits Auger characteristics due to the strong Coulomb coupling between electrons and holes. As a result, it does not conform to the Marcus inverted regime.^49^ However, interfacial electron transfer in the Marcus inverted regime can still take place from charge-separated states to surface-absorbed molecules, where the presence of strongly Coulomb-coupled holes is absent (Figure 5c).

**Figure 6:**
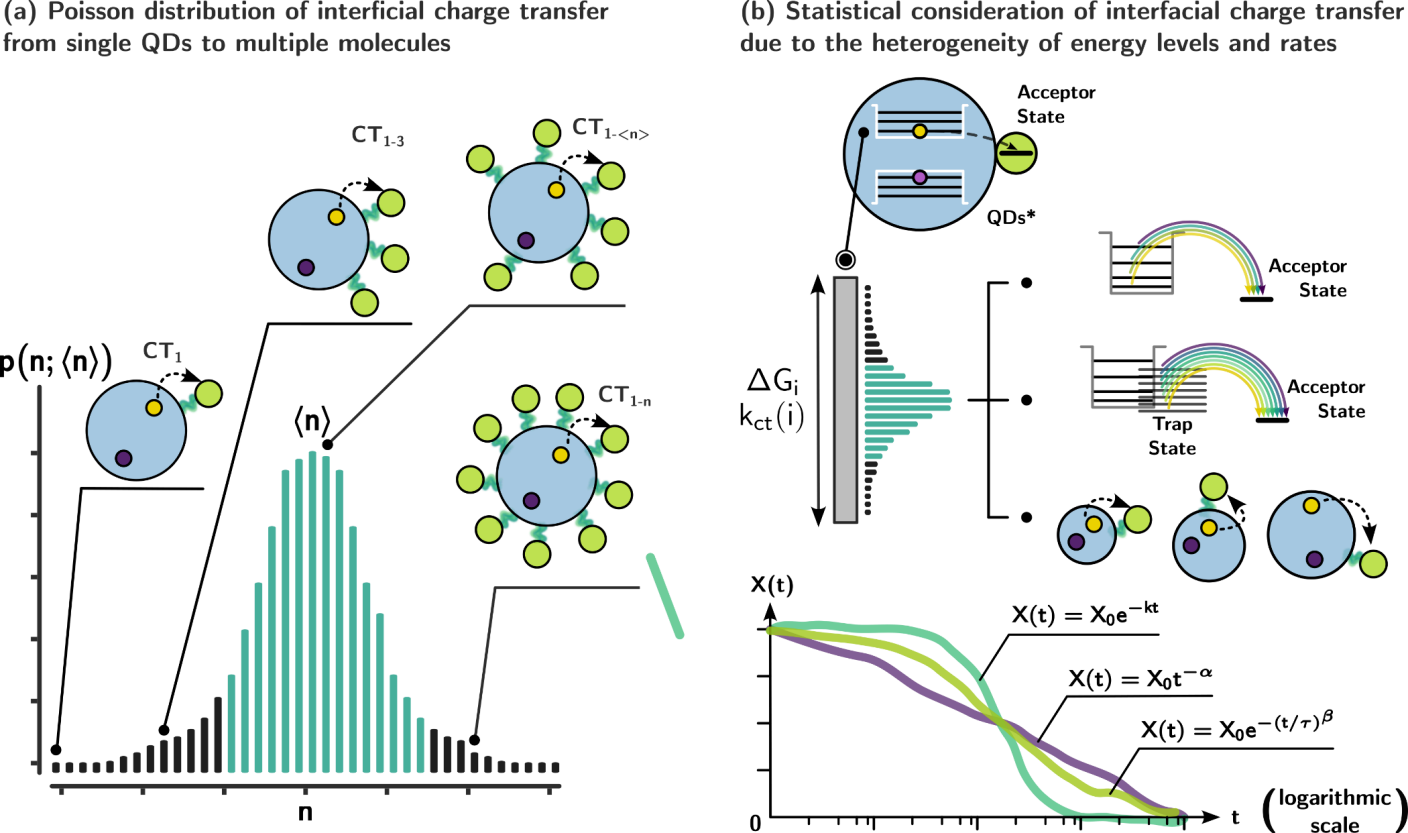
Schematic illustration of (a) Poisson distribution of molecules attached on QD surface and the direct interfacial charge transfer; (b) modified charge transfer kinetics with statistical consideration of the heterogeneity of rates, sites, and levels.

Moving beyond the treatment of QD–molecules as uniform and cohesive entities, it is important to acknowledge that multiple redox reactant molecules can attach to the surface of QDs, and this attachment can vary from dot to dot. Such complexity expands the potential of interfacial photoredox catalytic systems. Nevertheless, statistical knowledge can effectively address this challenge (Figure 6a). Assuming random chemisorption, the number of absorbed molecules per quantum dot must adhere to a Poisson distribution (eq 6):
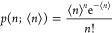
6where *p*(*n*; ⟨*n*⟩) describes the possibility of finding *n* molecules at the surface per QD, while the average number of attached molecules is ⟨*n*⟩.^50^ The corresponding electron transfer kinetics in such a system can be then described by the rate distribution equation (eq 7):
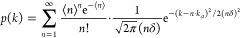
7where *k*_*CT*_ and δ stand for the average and standard deviation of the interfacial charge transfer rate in the 1:1 QD–molecule components, and the expected rate constant in a 1:*n* QD–molecule unit is therefore n*k_*CT*_.^51^ Notably, this phenomenon is linked to the chemisorption equilibrium, which offers a convenient method to determine the adsorption equilibrium constant within the QD–molecule complex.^52^ Another statistical concern arises when considering the impact of QDs’ inhomogeneity and energy levels on interfacial charge transfer (Figure 6b). This inhomogeneity leads to a broad distribution of charge transfer rates, resulting in an inability to accurately describe charge transfer and recombination using exponential decays. Unlike the conventional approach, which assumes fixed rate constants for each exponential component, a modified stretched exponential decay (eq 8) or power law decay (eq 9) must be employed to account for the wide distribution of single rates, enabling a more accurate representation of the time-resolved changes of the excited species *X*(*t*).^53,54^ In all cases, the analysis relies on the use of average charge transfer rates.
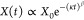
8
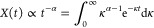
9

Based on the theoretical framework presented above, direct charge transfer at the interface between quantum dots (QDs) and molecules can be effectively modulated and manipulated. Alivisatos et al. demonstrated the ability to finely tune the morphology of QDs and molecular ligands, resulting in a photoinduced charge transfer rate that spans across 4 orders of magnitude.^55^ The diverse rate options empower researchers to fine-tune interfacial charge transfer processes, enabling a nuanced exploration of catalytic processes under varying conditions.

Moreover, Weiss et al. presented that the interfacial electron transfer rate from QDs to chemisorbed molecules can be controlled through the photoinduced isomerization reaction of the molecules. This phenomenon allows for the direct utilization of photoluminescent switching of QDs, as the photoinduced interfacial electron transfer acts as a significant competing channel to QDs’ emission, capable of being switched on or off.^56^ This symbiotic relationship can be strategically harnessed in the design and implementation of interfacial catalysis, offering a deep understanding of the complicated reaction process. Based on the preceding analysis, it is evident that the interfacial charge transfer occurring at the QD surface allows for the linkage between ultrafast light–matter interactions and relatively slower target redox reactions. The quantum confinement and interface morphology endow QDs with substantial application potential in photoredox catalysis. At the same time, the inherent complexity of the kinetics in these systems necessitates a comprehensive understanding for effective control and optimization. To achieve this goal, there is an urgent requirement for robust and sophisticated investigation tools capable of delving into the intricacies of interfacial processes.

## 3 Methods of Probing the Charge Kinetics at the Surface

To systematically capture and analyze intricate reaction networks with a high degree of precision, a comprehensive understanding of the fundamental mechanisms is harnessed by cutting-edge experimental methodologies, such as ultrafast spectroscopy and time-resolved microscopy, in conjunction with an advanced analytic algorithm, which can discern details of the reaction dynamics, predict reaction outputs, and achieve the final target of precise manipulation of the reactions. Combined studies with different types of time-resolved spectroscopy can cover the complete time window of the interfacial redox catalysis, contributing significantly to the broader comprehension of interfacial processes.^57,58^

**Figure 7:**
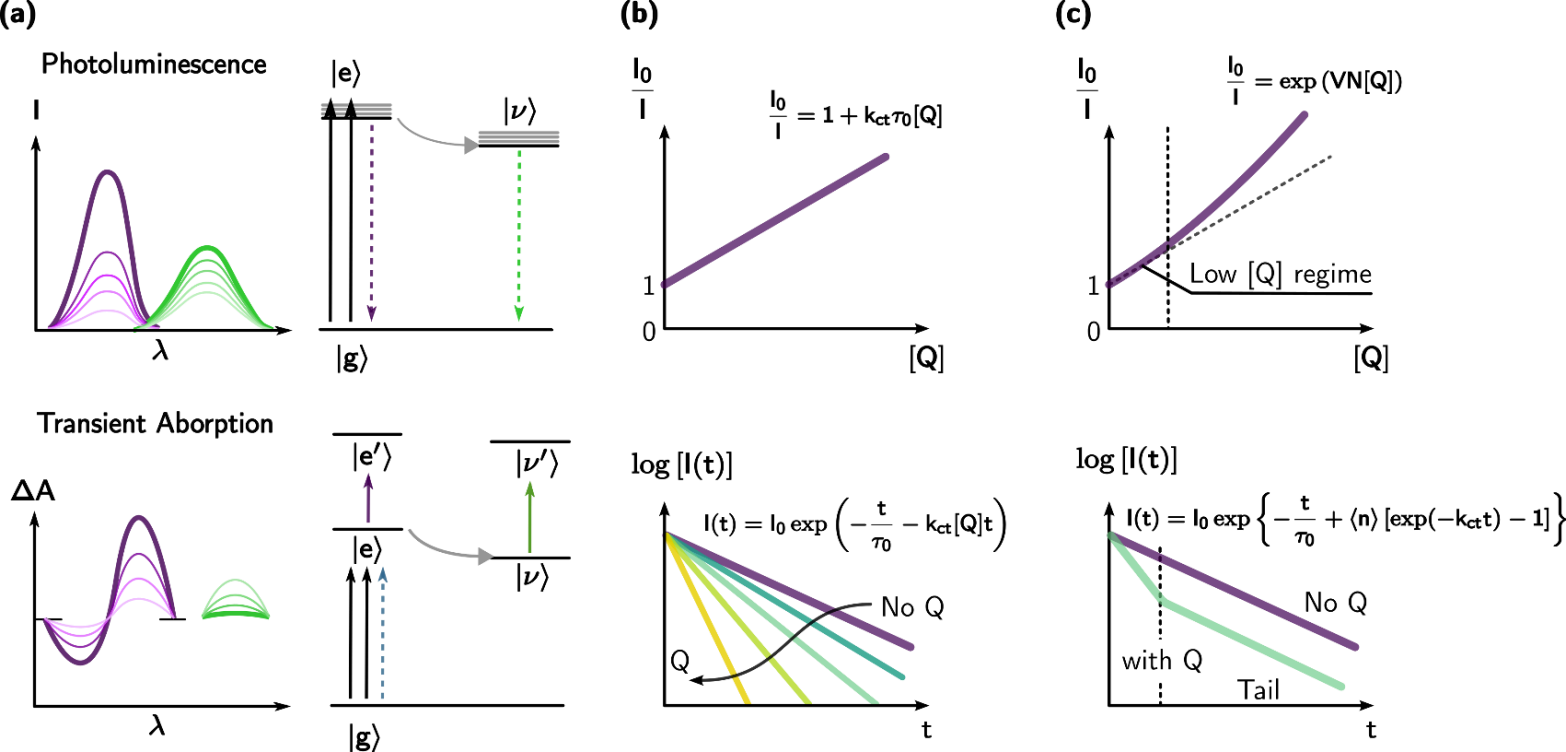
Schematic illustration of (a) the optical spectroscopy methods for investigating the interfacial charge transfer; (b) the kinetic behavior of collisional charge transfer at the QD–molecule interface; (c) the kinetic behavior of interfacial charge transfer from QDs to surface bound molecules.

The detection of photoinduced charge transfer kinetics between QDs and molecules commonly employs time-resolved spectroscopy, a straightforward and widely used method (Figure 7a). This approach is applicable to studying charge transfer at the QD–molecule interface. The second-order collisional charge transfer between QDs and molecular reactants follows the well-established Stern–Volmer relationship, which is frequently employed in the analysis of molecular photoredox catalysis (eq 10). The kinetic parameters of charge transfer can be determined through both steady-state and time-resolved emission experiments (Figure 7b).
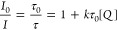
10

However, the surface chemistry of QDs introduces uncertainty in the photoinduced charge transfer kinetics at the QD–molecule interface. Direct charge transfer to surface-absorbed molecules can occur rapidly and efficiently, significantly contributing to the overall charge transfer processes. The direct electron transfer represents a first-order reaction with a rate constant measured in s^–1^. Nevertheless, it can still be affected by the local density of molecules due to statistical factors. In numerous cases, the nonlinear quenching of steady-state emission can be fitted using the exponential function proposed by Scaiano (eq 11), where the exponential factor *V* accounts for the volume effect of the molecular quencher and *N* is the Avogadro constant (Figure 7c).^59^
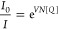
11

The observed phenomenon can be elucidated through the Poisson distribution theory (eq 6), where the likelihood of having no attached molecules and, consequently, no quenching effect is expressed as *p*(0; ⟨*n*⟩) = exp(−⟨*n*⟩). The emission intensity, associated with an average of ⟨*n*⟩ quenchers, is directly proportional to this probability, resulting in exponential quenching behavior (eq 11). When the concentration of the molecular reactant ([*Q*]) is low, the term exp(*VN*[*Q*]) approximates 1 + *VN*[*Q*], leading to quenching behavior similar to the Stern–Volmer effect (eq 10). However, caution is advised when interpreting “rate constants” that exceed the diffusion limit (approximately 10^10^ M^–1^ s^–1^ in most cases), as the validity of the Stern–Volmer relationship may be compromised, despite exhibiting a clear linear correlation with the reactant’s concentration.

In such scenarios, the actual interfacial electron transfer rate constant can be derived from measurements of the time-resolved emission quenching. The emission intensity over time (*I*(*t*)) is directly proportional to the density of the emissive excited state (*X*(*t*)). The overall decay of the emission can be treated as the superposition of decay profiles for each subsystem with the probability of each subsystem following the Poisson distribution (eq 12):
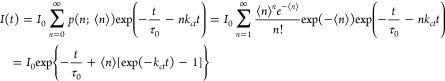
12The time-resolved emission equation, after a long time period, can be simplified as follows:
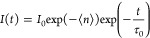
13From eq 13, it can be observed that the quenched emission decay of quantum dots (QDs) at the interface exhibits similar decay profiles to unquenched QDs at their tail part (Figure 7c).

Transient absorption spectroscopy is another crucial time-resolved technique for studying excited-state dynamics, particularly in the context of photoinduced charge transfer. Unlike emission spectroscopy, which primarily focuses on photoluminescent species like bandgap excitons in QDs, transient absorption spectroscopy offers a more comprehensive understanding by monitoring the disappearance of ground-state species, as well as the formation of photoinduced transformations. The key requirements are appropriate detection windows for both the temporal and spectral scales. However, compared to emission spectroscopy, transient absorption spectroscopy can be more intricate, as it can provide simultaneous information on multiple species. Nonetheless, the kinetic analysis algorithm for transient absorption spectroscopy shares similarities with time-resolved emission spectroscopy (eqs 10–13). The transient absorption amplitude (Δ*A*) follows a linear relationship with the concentration of the excited species (eq 14):
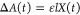
14where the molar absorption coefficient (ε) and the length of the light path for the probing beam (*l*) remain constant throughout the measurements. Thus, the transient absorption signal serves as another linear indicator for tracking kinetics in charge transfer and photoredox processes and can provide the kinetic profile of possible intermediate states in the photocatalytic cycle. Advanced spectroscopic techniques can also be employed to investigate the interfacial charge transfer. Time-resolved terahertz spectroscopy (TRTS), for instance, offers a convenient means to measure the kinetics of hot carriers in QDs by providing information on local alternating current conductivity.^60^ This method is particularly useful when conventional transient absorption is limited in its applicability. Nonlinear spectroscopy, such as second harmonic generation (SHG) and sum frequency generation (SFG), exhibits high sensitivity to interface properties and can be employed to explore the fundamental photophysics of interactions at the surface of QDs.^61,62^

Besides the optical spectroscopy, X-ray spectroscopy serves as a valuable complement to optical spectroscopy in exploring photoinduced interactions at interfaces. Specifically, X-ray photoelectron spectroscopy (XPS) and X-ray absorption spectroscopy (XAS) offer direct and detailed insights into the electronic structures of intermediate species involved in photocatalytic redox, based on distinct physical principles. The rearrangement of electrons among atoms, resulting from photoinduced charge transfer, can be precisely captured using X-ray spectroscopic methods under light conditions.^63^ XPS proves to be an effective surface analysis technique, enabling the characterization of the elemental compositions, chemical states, and electronic structures. Consequently, this is highly suitable for investigating charge transfer at interfaces. Bi et al. have developed reliable methodology utilizing in situ irradiation XPS to monitor interfacial charge transfer.^64,65^ By combining angle-resolved XPS techniques with photon excitation, scientists can reveal depth-resolved information about the chemical structure at the interface, thus providing detailed insights into interfacial charge transfer.

**Figure 8:**
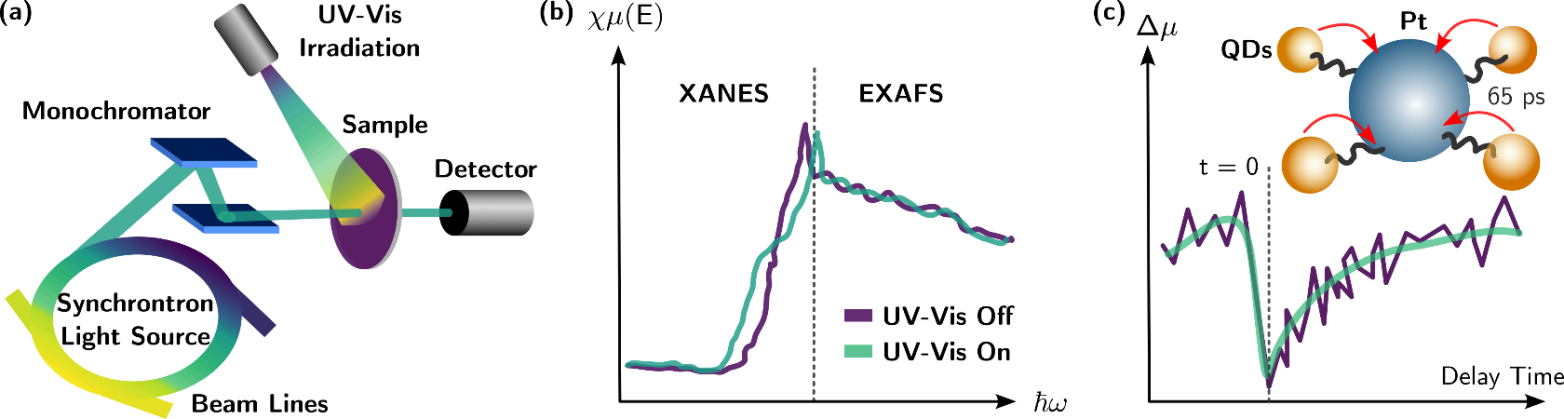
Schematic illustration of (a) in situ irradiation XAS for investigating the interfacial charge transfer; (b) the typical spectra of XAS with and without irradiation for interfacial charge transfer; (c) the representative case of time-resolved XAS for revealing interfacial charge transfer, adapted with permission from ref (25), Copyright 2017 American Chemical Society.

On the other hand, XAS excels at detecting valence states, orbital hybridization, and coordination environments. Consequently, it also serves as an effective method for investigating the interfacial charge transfer. By comparing XAS spectra with and without photon irradiation, it is possible to identify potential chemical shifts resulting from interfacial charge transfer (Figure 8a).^66^ In addition to these in situ techniques, time-resolved X-ray spectroscopy offers time-resolved information about interfacial charge transfer at short time scales (Figure 8b). This progress is primarily attributed to synchronous radiation centers or X-ray free-electron lasers (XFELs). Pioneering studies conducted at Argonne National Laboratory have demonstrated the effectiveness of X-ray transient absorption spectroscopy as a powerful tool for investigating the kinetics of rapid chemical transformation and interfacial charge transfer.^67−69^ Our team was the first to utilize this technique to observe ultrafast charge transfer between QDs and cocatalysts, demonstrating direct charge transfer at the QD interface at the picosecond level, which surpasses diffusive charge transfer (Figure 8c).^25^

Additionally, other characterization methods can be employed to study the interfacial charge transfer. Li and Fan developed a methodology utilizing surface photovoltage microscopy (SPV) to monitor charge migration in both space and time.^70^ This approach offers an imaging perspective, providing significant advantages in the study of interfacial charge transfer.^71^ Cossairt et al. demonstrated the utility of electrochemical methods in providing essential parametric information about interfacial charge transfer.^72^ For instance, cyclic voltammetry can determine the rate-limiting step in interfacial photoredox reactions. Significant advancements have been made in both instrumental and applied research related to the photophysical and photochemical properties, such as charge transfer and separation dynamics at the interface.^73^ In tandem with advancements in instrumental science, the integration of data science stands as a pivotal facilitator in the mechanistic exploration of interfacial redox catalysis. Capitalizing on sophisticated techniques such as big data analysis and machine learning, scientists can systematically unveil the intricacies concealed within the complex reaction networks associated with interfacial photoredox processes.^74,75^ Through the discerning analysis of vast data sets, encompassing intricate intermolecular interactions and dynamic reaction kinetics, researchers gain a comprehensive understanding of the mechanistic underpinnings governing interfacial redox catalysis. These developments have greatly aided in understanding the mechanistic investigation of photocatalytic reactions and hold great potential for future research on the mechanisms of photoredox catalysis at complex levels.

## 4 Interfacial Charge Transfer in Photoredox Catalysis

Interfacial photoredox catalysis presents distinctive advantages, owing to the unique characteristics of the QD–molecular interface. The precise tunability of QDs allows for tailored absorption properties, enabling efficient utilization of solar energy for photoredox reactions. This specificity in light absorption, coupled with the ability to modulate electron transfer processes at the interface, distinguishes interfacial photoredox catalysis from conventional catalytic systems. Such control is particularly advantageous in the synthesis of complex organic compounds, where selectivity and precision are paramount. The investigation began with the exploration of straightforward reactions, such as the photoinduced decomposition of organic compounds.^77,78^ Researchers soon recognized that the intricacies of the QDs’ interface caused these reactions to deviate from traditional solution-phase models, thereby presenting fresh opportunities for organic synthesis. The interfacial charge transfer process provides a unique platform for the design and manipulation of target organic transformations. Direct cross coupling at the interface is, therefore, considered as the most promising model reaction. In 2014, our group demonstrated S–S cross coupling at the surface of colloidal CdSe QDs, transforming thiols into disulfides accompanied by hydrogen evolution.^79^ The formation of disulfides is a pivotal biochemical process essential for constructing the three-dimensional structure of proteins. When compared with homogeneous catalytic systems, the QD–molecular interface was found to enhance rapid charge transfer and provide unique reaction sites. Thiol radicals formed on the QDs surface, significantly increasing the likelihood of their encounter and subsequent coupling. Subsequently exposed metal atoms could serve as cocatalysts for subsequent proton-to-hydrogen transformations.^37,80^ Another important case of mild reactions was revealed in 2017, when König employed ZnSe/CdS core/shell QDs as photocatalysts, enabling the formation of carbon–carbon bonds from aryl halides.^81^ This type of reaction holds significant relevance for the pharmaceutical and fine chemicals industry. The interface of these QDs creates an optimal environment for coupling reactions, surpassing the performance of traditional organic dyes and metal complexes. These reactions exemplify the regulation of photoredox transformations through interface charge transfer and spatially confined reactions. At the interface, QD–molecule hybrid photocatalysts exhibit extraordinary performance and greatly reduce the harsh requirements in coupling reactions. The rapid and efficient interfacial charge transfer at an individual light-harvesting unit of QDs guarantees successful initiation of the redox reaction cycle.

**Figure 9:**
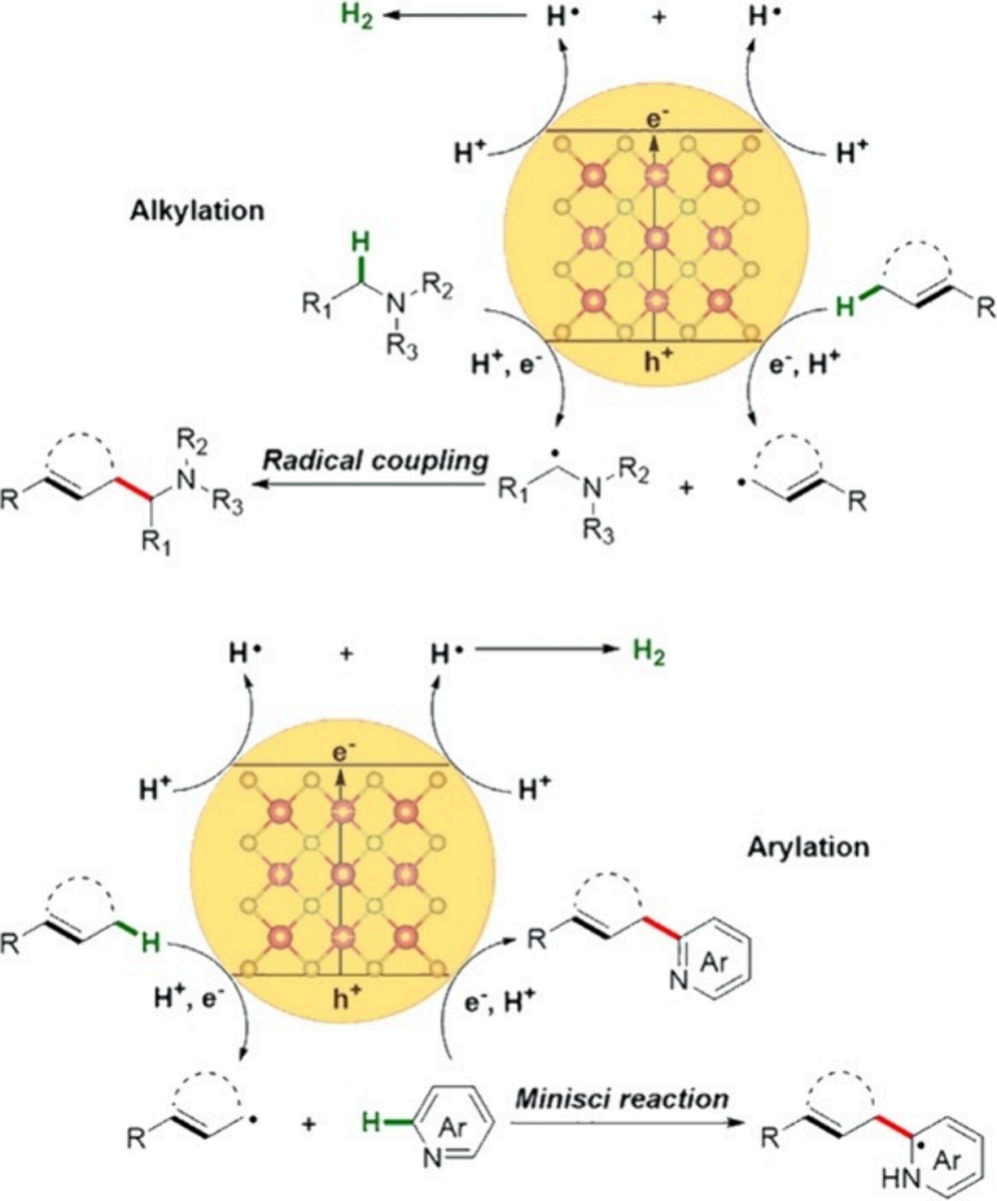
Schematic illustration of photocatalytic C–C cross coupling at the interface of QDs, adapted with permission from ref (76), Copyright 2021 Elsevier.

The approach centers on interfacial photoredox strategies and capitalizes on the distinctive characteristics of semiconductors to enable environmentally friendly hydrogen evolution integrated within photocatalytic cross-coupling processes at the QDs’ interface. Our group has dedicated efforts to address intricate redox reactions traditionally necessitating rigorous conditions and complex synthetic methodologies.^82,83^ Detailed studies on the reaction mechanism revealed that, for example, allylic radicals and thiyl radicals, generated on the QDs’ surface, facilitate C–S bond formation without the need for external oxidants or radical initiators. The ability of QD photocatalysts to perform C–P cross-coupling and C–N cross-coupling via analogous interfacial photoredox strategies has been revealed.^84,85^ The in-depth exploration of QDs’ photophysics, especially at the interface, led to the first instance of direct alkylation and arylation of allylic C(SP^3^)-H bonds, powered by solar energy and accompanied by H_2_ evolution (Figure 9).^76^ This H_2_ evolution cross-coupling is a crucial step forward in atomic economy.^86,87^ The interface of CdSe QDs offers exceptional sites for activating hydrocarbons and promoting the coupling of radical intermediates. The activation of the C(SP^3^)-H bond in tetrahydrofuran (THF) achieves site-selective C–C cross-coupling at the CdSe QD interface.^88^ Recently, amine-free directing group free α-C–H alkylation of cyclic ketones under visible light irradiation forges a novel pathway for α-C–H functionalization within carbonyl chemistry, as a complement to the traditional amine catalysis with directing group.^89^ Compared to the homogeneous photoredox catalysis, reactions at the QD interface offer substantial advantages in terms of reactant activation and directed catalysis. The unique characteristics of the QD interface facilitate tailored interactions with reactants, leading to the precise activation of specific functional groups.

The fast interfacial charge transfer can be involved in building the multiscale charge transfer channel with high efficiency and stability, which greatly promotes the redox transformations. The interplay of reactants, driven by forces such as electrostatic attraction, results in quasi-static donor–acceptor complexes within these assemblies. This affects the charge transfer kinetics, deviating from the traditional Stern–Volmer relationship.^90^ An ultrafast charge transfer at the interface supersedes diffusion, introducing a novel channel from the homogeneous catalysis.^91^

**Figure 10:**
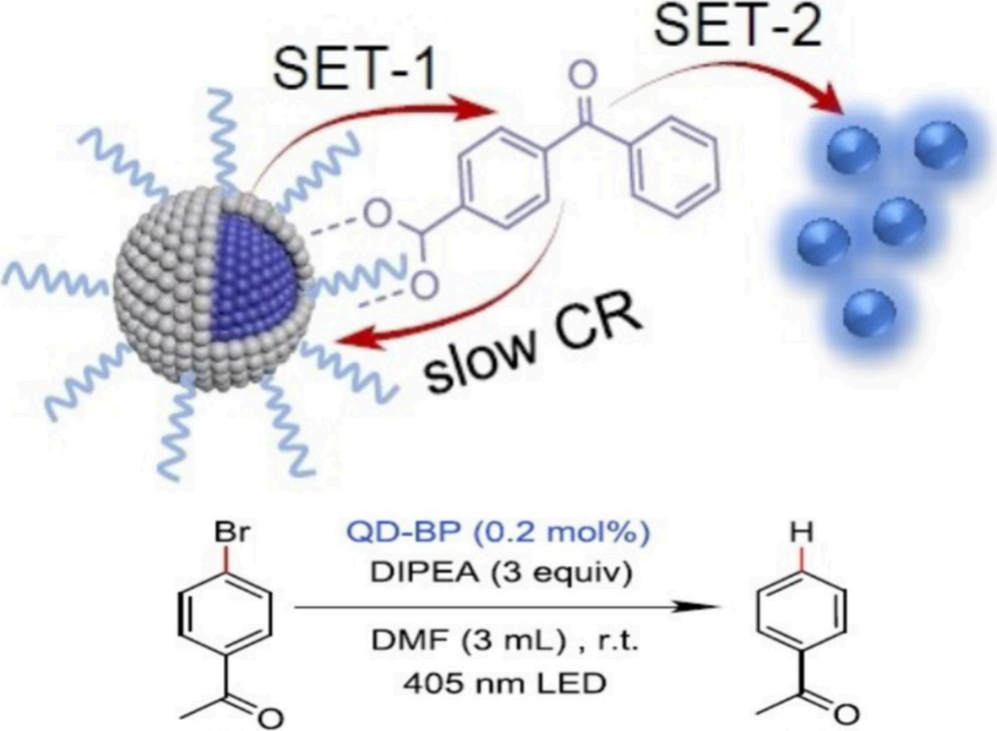
Schematic illustration of the photoreduction of aryl bromides by the consecutive photoinduced electron transfer from QD photocatalyst, adapted with permission from ref (92). Copyright 2022 Wiley.

Building upon this kinetic relationship, Wu et al. devised ZnSe/ZnS QDs as photocatalysts and achieved highly efficient photoreduction of aryl bromides (Figure 10).^92^ The interfacial electron transfer exhibits a lifetime of 13.4 ps from the QDs to the chemisorbed ligands, resulting in a long-lived charge separation state with a lifetime of up to 440 ns. This charge separation is subsequently followed by a second-step single transfer to the reactant aryl bromides. Thus, interfacial charge transfer enables the intricate design of consecutive electron transfers, promoting QDs’ efficacy in photoredox catalysis. The reactivity can be finely tuned through surface modification. Weiss et al. presented a notable example of regulating interfacial charge transfer properties by doping fluorodecanethiol into the oleate ligand shell of PbS QDs, influencing the permeability of reactants through the QD shell.^93^ The photocatalytic behavior of QDs can be modified by selectively exposing and loading them on their surface, and the surface properties offer vast potential for QD fine-tuning, catering to specific organic transformations.^94^ Exploiting the advantages of QDs in catalysis transcends the conventional limitations of homogeneous systems, offering a promising avenue for advancing the efficiency and selectivity of photoredox processes in a diverse array of chemical transformations.

The quantum confinement effects and size-dependent electronic structures of QDs and the multiple level energy diagram play pivotal roles in creating a reaction environment conducive to overcoming thermodynamic barriers. Krauss and Weix demonstrated a remarkable example of driving interfacial photoredox reactions with high thermodynamic demand by the Auger-enabled photoredox process at the QD surface.^95^ The hot electrons generated by the Auger process of QDs provide an extremely high reductive potential for hydrodechlorination reactions. Compared to traditional molecular reducing agents, this type of reaction exhibits both high activity and high stability simultaneously. Weiss et al. provided strategic ligand design that promotes the delocalization and capture of excitons in QDs, fostering interfacial charge transfer from biexcitonic states to molecular reactants, thus facilitating efficient multielectron photoredox catalysis.^96^ They also proposed a general coupled multicharge transfer mechanism at the QD–molecule interface, realizing intricate multicharge transfer reactions, with pronounced orthogonality and selectivity.^97,98^ Recently, Krauss, Weix, and co-workers achieved highly efficient cross-electrophile coupling of the C(*sp*^3^)–C(*sp*^2^) bond in the hybrid system of CdS QDs and metallaphotoredox cocatalyst.^99^ In terms of the interfacial coupling mechanism, Chen reported the first case of stereoselective C–C oxidative dimerization using perovskite QDs.^100^ The excellent stereoselectivities (>99%) of the dl-isomer indicate the potential for applications in fine chemical synthesis. How the unique morphology and configuration lead to intricate mechanistic pathways has become a recent hot topic in interfacial photoredox. Solving such a puzzle enables the exquisite control of reaction outcomes. The distinct capability not only showcases the versatility of QDs in catalysis but also positions them as a valuable tool for advancing the understanding and application of the promising and challenging reactions in the field of chemical synthesis.

## 5 Summary

Interfacial charge transfer, spanning mechanisms and time scales from ultrafast to slow regimes, plays a crucial role in photoredox catalysis.^101^ Determining the rate constant of individual redox steps helps us outline how photogenerated charges are formed and consumed. This sheds light on the primary mechanisms behind photoredox catalysis and explains the dominance of major products over other reaction channels on heterogeneous photocatalysts’ surfaces. Integrating fundamental mathematical models with advanced spectroscopic techniques allows for real-time monitoring, analysis, and simulation of electron transfer. This framework aids scientists in comprehending and manipulating intricate photoredox reactions such as cross-coupling and inert bond cleavage. These redox reactions form the foundation of modern synthesis, paving the way for the precise fabrication of functional materials for energy, environmental, and pharmacological applications.

Herein, we introduced interfacial charge transfer and photoredox by the representative model of QD–molecule hybrid systems. Molecular reactants, when interacting with QDs, can undergo diffusion and be activated by interfacial charge transfer, effectively using QDs as molecular photocatalysts. Through meticulous design, charge separation can supersede the charge recombination pathways. The Stern–Volmer equation (eq 10) can be employed to determine kinetics, optimizing collisional charge transfer at the QD interface for maximum photoredox catalysis reactivity.^85,102^ Proper control and understanding of these processes are imperative for achieving specific redox transformations. This includes adopting strategies to manipulate interfacial charge transfer rates and selectivity, creating novel materials, and introducing catalysts that boost efficient charge separation and transport. Bridging molecular engineering, surface science, and material synthesis is essential to craft interfaces that potentiate preferred redox routes and enhance photoredox catalysis efficiency.

In summary, interfacial charge transfer stands out as the determining step, guiding the overarching reaction and exhibiting unique behaviors. Though this process can be more intricate than homogeneous molecular systems, its complexity offers rich design possibilities for intricate transformations, as recent applications have demonstrated. However, the exploration of interfacial charge transfer in photoredox catalysis is far from exhaustive. Given its intricacy and significance, a deeper, holistic grasp of this process is vital. This calls for more in-depth probing into the photophysics of interfacial charge transfer, its dynamics, and the influencing factors. The advent of experimental techniques, such as ultrafast spectroscopy and time-resolved microscopy, alongside theoretical modeling can grant a holistic understanding. Combining these with computational methods, such as quantum chemical calculations and molecular dynamics simulations, can offer invaluable insights. Machine learning and artificial intelligence can further refine the predictions and designs of photocatalytic systems. As research progresses, interdisciplinary collaboration and knowledge exchange among scientists from diverse fields, such as chemistry, materials science, physics, and computational science, will be crucial for tackling the multifaceted challenges associated with interfacial charge transfer and photoredox catalysis. By fostering collaboration and sharing expertise, researchers can push the boundaries of knowledge and accelerate the development of innovative approaches for sustainable energy conversion, environmental remediation, and organic synthesis. With confidence in the promising future of interface-regulated photoredox catalysis, we have just begun the journey of discovery and advancement has just begun.

## References

[ref1] Lu H.; Huang Z.; Martinez M. S.; Johnson J. C.; Luther J. M.; Beard M. C. Transforming energy using quantum dots. Energy Environ. Sci. 2020, 13 (5), 1347–1376. 10.1039/C9EE03930A.

[ref2] Ye C.; Zhang D.-S.; Chen B.; Tung C.-H.; Wu L.-Z. Quantum dots: Another choice to sensitize organic transformations. Chem. Phys. Rev. 2023, 4 (1), 011304 10.1063/5.0126893.

[ref3] Saniepay M.; Mi C.; Liu Z.; Abel E. P.; Beaulac R. Insights into the Structural Complexity of Colloidal CdSe Nanocrystal Surfaces: Correlating the Efficiency of Nonradiative Excited-State Processes to Specific Defects. J. Am. Chem. Soc. 2018, 140 (5), 1725–1736. 10.1021/jacs.7b10649. 29293359

[ref4] Li X.-B.; Tung C.-H.; Wu L.-Z. Semiconducting quantum dots for artificial photosynthesis. Nat. Rev. Chem. 2018, 2 (8), 160–173. 10.1038/s41570-018-0024-8.

[ref5] Harris C.; Kamat P. V. Photocatalysis with CdSe Nanoparticles in Confined Media: Mapping Charge Transfer Events in the Subpicosecond to Second Timescales. ACS Nano 2009, 3 (3), 682–690. 10.1021/nn800848y. 19226135

[ref6] Burke R.; Bren K. L.; Krauss T. D. Semiconductor nanocrystal photocatalysis for the production of solar fuels. J. Chem. Phys. 2021, 154 (3), 030901 10.1063/5.0032172. 33499632

[ref7] Bai S.; Jiang J.; Zhang Q.; Xiong Y. Steering charge kinetics in photocatalysis: intersection of materials syntheses, characterization techniques and theoretical simulations. Chem. Soc. Rev. 2015, 44 (10), 2893–2939. 10.1039/C5CS00064E. 25904385

[ref8] Meng S.-L.; Ye C.; Li X.-B.; Tung C.-H.; Wu L.-Z. Photochemistry Journey to Multielectron and Multiproton Chemical Transformation. J. Am. Chem. Soc. 2022, 144 (36), 16219–16231. 10.1021/jacs.2c02341. 36054091

[ref9] Harris R. D.; Bettis Homan S.; Kodaimati M.; He C.; Nepomnyashchii A. B.; Swenson N. K.; Lian S.; Calzada R.; Weiss E. A. Electronic Processes within Quantum Dot-Molecule Complexes. Chem. Rev. 2016, 116 (21), 12865–12919. 10.1021/acs.chemrev.6b00102. 27499491

[ref10] Wu X.; Fan X.; Xie S.; Lin J.; Cheng J.; Zhang Q.; Chen L.; Wang Y. Solar energy-driven lignin-first approach to full utilization of lignocellulosic biomass under mild conditions. Nature Catalysis 2018, 1 (10), 772–780. 10.1038/s41929-018-0148-8.

[ref11] Weng B.; Qi M.-Y.; Han C.; Tang Z.-R.; Xu Y.-J. Photocorrosion Inhibition of Semiconductor-Based Photocatalysts: Basic Principle, Current Development, and Future Perspective. ACS Catal. 2019, 9 (5), 4642–4687. 10.1021/acscatal.9b00313.

[ref12] Jiang Y.; Weiss E. A. Colloidal Quantum Dots as Photocatalysts for Triplet Excited State Reactions of Organic Molecules. J. Am. Chem. Soc. 2020, 142 (36), 15219–15229. 10.1021/jacs.0c07421. 32810396

[ref13] Han Y.; He S.; Wu K. Molecular Triplet Sensitization and Photon Upconversion Using Colloidal Semiconductor Nanocrystals. ACS Energy Lett. 2021, 6 (9), 3151–3166. 10.1021/acsenergylett.1c01348.

[ref14] Peterson M. D.; Cass L. C.; Harris R. D.; Edme K.; Sung K.; Weiss E. A. The Role of Ligands in Determining the Exciton Relaxation Dynamics in Semiconductor Quantum Dots. Annu. Rev. Phys. Chem. 2014, 65 (1), 317–339. 10.1146/annurev-physchem-040513-103649. 24364916

[ref15] Knowles K. E.; Malicki M.; Weiss E. A. Dual-Time Scale Photoinduced Electron Transfer from PbS Quantum Dots to a Molecular Acceptor. J. Am. Chem. Soc. 2012, 134 (30), 12470–12473. 10.1021/ja3060222. 22813233

[ref16] Boulesbaa A.; Issac A.; Stockwell D.; Huang Z.; Huang J.; Guo J.; Lian T. Ultrafast Charge Separation at CdS Quantum Dot/Rhodamine B Molecule Interface. J. Am. Chem. Soc. 2007, 129 (49), 15132–15133. 10.1021/ja0773406. 18001027

[ref17] Klimov V. I. Spectral and Dynamical Properties of Multiexcitons in Semiconductor Nanocrystals. Annu. Rev. Phys. Chem. 2007, 58 (1), 635–673. 10.1146/annurev.physchem.58.032806.104537. 17163837

[ref18] Yang Y.; Rodríguez-Córdoba W.; Lian T. Ultrafast Charge Separation and Recombination Dynamics in Lead Sulfide Quantum Dot-Methylene Blue Complexes Probed by Electron and Hole Intraband Transitions. J. Am. Chem. Soc. 2011, 133 (24), 9246–9249. 10.1021/ja2033348. 21615168

[ref19] Dutta P.; Tang Y.; Mi C.; Saniepay M.; McGuire J. A.; Beaulac R. Ultrafast hole extraction from photoexcited colloidal CdSe quantum dots coupled to nitroxide free radicals. J. Chem. Phys. 2019, 151 (17), 174706 10.1063/1.5124887. 31703504

[ref20] Lian S.; Weinberg D. J.; Harris R. D.; Kodaimati M. S.; Weiss E. A. Subpicosecond Photoinduced Hole Transfer from a CdS Quantum Dot to a Molecular Acceptor Bound Through an Exciton-Delocalizing Ligand. ACS Nano 2016, 10 (6), 6372–6382. 10.1021/acsnano.6b02814. 27281685

[ref21] Eliasson N.; Rimgard B. P.; Castner A.; Tai C.-W.; Ott S.; Tian H.; Hammarström L. Ultrafast Dynamics in Cu-Deficient CuInS2 Quantum Dots: Sub-Bandgap Transitions and Self-Assembled Molecular Catalysts. J. Phys. Chem. C 2021, 125 (27), 14751–14764. 10.1021/acs.jpcc.1c02468.

[ref22] Huang J.; Stockwell D.; Huang Z.; Mohler D. L.; Lian T. Photoinduced Ultrafast Electron Transfer from CdSe Quantum Dots to Re-bipyridyl Complexes. J. Am. Chem. Soc. 2008, 130 (17), 5632–5633. 10.1021/ja8003683. 18393497

[ref23] Sandroni M.; Gueret R.; Wegner K. D.; Reiss P.; Fortage J.; Aldakov D.; Collomb M. N. Cadmium-free CuInS2/ZnS quantum dots as efficient and robust photosensitizers in combination with a molecular catalyst for visible light-driven H2 production in water. Energy Environ. Sci. 2018, 11 (7), 1752–1761. 10.1039/C8EE00120K.

[ref24] Tvrdy K.; Frantsuzov P. A.; Kamat P. V. Photoinduced electron transfer from semiconductor quantum dots to metal oxide nanoparticles. Proc. Natl. Acad. Sci. U. S. A 2011, 108 (1), 29–34. 10.1073/pnas.1011972107. 21149685 PMC3017152

[ref25] Li X.-B.; Gao Y.-J.; Wang Y.; Zhan F.; Zhang X.-Y.; Kong Q.-Y.; Zhao N.-J.; Guo Q.; Wu H.-L.; Li Z.-J.; Tao Y.; Zhang J.-P.; Chen B.; Tung C.-H.; Wu L.-Z. Self-Assembled Framework Enhances Electronic Communication of Ultrasmall-Sized Nanoparticles for Exceptional Solar Hydrogen Evolution. J. Am. Chem. Soc. 2017, 139 (13), 4789–4796. 10.1021/jacs.6b12976. 28281343

[ref26] Wu K.; Li Q.; Du Y.; Chen Z.; Lian T. Ultrafast exciton quenching by energy and electron transfer in colloidal CdSe nanosheet-Pt heterostructures. Chem. Sci. 2015, 6 (2), 1049–1054. 10.1039/C4SC02994A. 29560193 PMC5811111

[ref27] Tisdale W. A.; Williams K. J.; Timp B. A.; Norris D. J.; Aydil E. S.; Zhu X.-Y. Hot-Electron Transfer from Semiconductor Nanocrystals. Science 2010, 328 (5985), 1543–1547. 10.1126/science.1185509. 20558714

[ref28] Singh R.; Liu W.; Lim J.; Robel I.; Klimov V. I. Hot-electron dynamics in quantum dots manipulated by spin-exchange Auger interactions. Nat. Nanotechnol. 2019, 14 (11), 1035–1041. 10.1038/s41565-019-0548-1. 31591527

[ref29] Schleusener A.; Micheel M.; Benndorf S.; Rettenmayr M.; Weigand W.; Wächtler M. Ultrafast Electron Transfer from CdSe Quantum Dots to an [FeFe]-Hydrogenase Mimic. J. Phys. Chem. Lett. 2021, 12 (18), 4385–4391. 10.1021/acs.jpclett.1c01028. 33939438

[ref30] Mondal N.; Samanta A. Ultrafast Charge Transfer and Trapping Dynamics in a Colloidal Mixture of Similarly Charged CdTe Quantum Dots and Silver Nanoparticles. J. Phys. Chem. C 2016, 120 (1), 650–658. 10.1021/acs.jpcc.5b08630.

[ref31] Okuhata T.; Katayama T.; Tamai N. Ultrafast and Hot Electron Transfer in CdSe QD-Au Hybrid Nanostructures. J. Phys. Chem. C 2020, 124 (1), 1099–1107. 10.1021/acs.jpcc.9b09042.

[ref32] Singhal P.; Ghosh H. N. Hot-Hole Extraction from Quantum Dot to Molecular Adsorbate. Chem.—Eur. J. 2015, 21 (11), 4405–4412. 10.1002/chem.201405947. 25656635

[ref33] Jiang Z.-J.; Kelley D. F. Hot and Relaxed Electron Transfer from the CdSe Core and Core/Shell Nanorods. J. Phys. Chem. C 2011, 115 (11), 4594–4602. 10.1021/jp112424z.

[ref34] Das S.; Rakshit S.; Datta A. Interplay of Multiexciton Relaxation and Carrier Trapping in Photoluminescent CdS Quantum Dots Prepared in Aqueous Medium. J. Phys. Chem. C 2020, 124 (51), 28313–28322. 10.1021/acs.jpcc.0c09366.

[ref35] Kuehnel M. F.; Sahm C. D.; Neri G.; Lee J. R.; Orchard K. L.; Cowan A. J.; Reisner E. ZnSe quantum dots modified with a Ni(cyclam) catalyst for efficient visible-light driven CO2 reduction in water. Chem. Sci. 2018, 9 (9), 2501–2509. 10.1039/C7SC04429A. 29732127 PMC5911736

[ref36] Wu K.; Du Y.; Tang H.; Chen Z.; Lian T. Efficient Extraction of Trapped Holes from Colloidal CdS Nanorods. J. Am. Chem. Soc. 2015, 137 (32), 10224–10230. 10.1021/jacs.5b04564. 26221916

[ref37] Gao Y.-J.; Li X.-B.; Wu H.-L.; Meng S.-L.; Fan X.-B.; Huang M.-Y.; Guo Q.; Tung C.-H.; Wu L.-Z. Exceptional Catalytic Nature of Quantum Dots for Photocatalytic Hydrogen Evolution without External Cocatalysts. Adv. Funct. Mater. 2018, 28 (33), 1801769 10.1002/adfm.201801769.

[ref38] Liu E.; Zhu H.; Yi J.; Kobbekaduwa K.; Adhikari P.; Liu J.; Shi Y.; Zhang J.; Li H.; Oprisan A.; Rao A. M.; Sanabria H.; Chen O.; Gao J. Manipulating Charge Transfer from Core to Shell in CdSe/CdS/Au Heterojunction Quantum Dots. ACS Appl. Mater. Interfaces 2019, 11 (51), 48551–48555. 10.1021/acsami.9b17339. 31782302 PMC7325308

[ref39] Luo X.; Liang G.; Wang J.; Liu X.; Wu K. Picosecond multi-hole transfer and microsecond charge-separated states at the perovskite nanocrystal/tetracene interface. Chem. Sci. 2019, 10 (8), 2459–2464. 10.1039/C8SC04408B. 30881674 PMC6385846

[ref40] Young R. M.; Jensen S. C.; Edme K.; Wu Y.; Krzyaniak M. D.; Vermeulen N. A.; Dale E. J.; Stoddart J. F.; Weiss E. A.; Wasielewski M. R.; Co D. T. Ultrafast Two-Electron Transfer in a CdS Quantum Dot-Extended-Viologen Cyclophane Complex. J. Am. Chem. Soc. 2016, 138 (19), 6163–6170. 10.1021/jacs.5b13386. 27111529

[ref41] Zhu H.; Yang Y.; Hyeon-Deuk K.; Califano M.; Song N.; Wang Y.; Zhang W.; Prezhdo O. V.; Lian T. Auger-Assisted Electron Transfer from Photoexcited Semiconductor Quantum Dots. Nano Lett. 2014, 14 (3), 1263–1269. 10.1021/nl4041687. 24359156

[ref42] Hyeon-Deuk K.; Kim J.; Prezhdo O. V. Ab Initio Analysis of Auger-Assisted Electron Transfer. J. Phys. Chem. Lett. 2015, 6 (2), 244–249. 10.1021/jz502505m. 26263457

[ref43] Haiming Z.; Ye Y.; Nianhui S.; William R.-C.; Tianquan L. Controlling interfacial charge separation and recombination dynamics in QDs by wave function engineering. Proc. SPIE 2011, 809802 10.1117/12.892873.

[ref44] Reynal A.; Willkomm J.; Muresan N. M.; Lakadamyali F.; Planells M.; Reisner E.; Durrant J. R. Distance dependent charge separation and recombination in semiconductor/molecular catalyst systems for water splitting. Chem. Commun. 2014, 50 (84), 12768–12771. 10.1039/C4CC05143B. PMC4183993 25207748

[ref45] Zhu H.; Song N.; Lian T. Controlling Charge Separation and Recombination Rates in CdSe/ZnS Type I Core-Shell Quantum Dots by Shell Thicknesses. J. Am. Chem. Soc. 2010, 132 (42), 15038–15045. 10.1021/ja106710m. 20925344

[ref46] Wilker M. B.; Utterback J. K.; Greene S.; Brown K. A.; Mulder D. W.; King P. W.; Dukovic G. Role of Surface-Capping Ligands in Photoexcited Electron Transfer between CdS Nanorods and [FeFe] Hydrogenase and the Subsequent H2 Generation. J. Phys. Chem. C 2018, 122 (1), 741–750. 10.1021/acs.jpcc.7b07229.

[ref47] Morris-Cohen A. J.; Peterson M. D.; Frederick M. T.; Kamm J. M.; Weiss E. A. Evidence for a Through-Space Pathway for Electron Transfer from Quantum Dots to Carboxylate-Functionalized Viologens. J. Phys. Chem. Lett. 2012, 3 (19), 2840–2844. 10.1021/jz301318m.

[ref48] Olshansky J. H.; Ding T. X.; Lee Y. V.; Leone S. R.; Alivisatos A. P. Hole Transfer from Photoexcited Quantum Dots: The Relationship between Driving Force and Rate. J. Am. Chem. Soc. 2015, 137 (49), 15567–15575. 10.1021/jacs.5b10856. 26597761

[ref49] Wang J.; Ding T.; Gao K.; Wang L.; Zhou P.; Wu K. Marcus inverted region of charge transfer from low-dimensional semiconductor materials. Nat. Commun. 2021, 12 (1), 6333 10.1038/s41467-021-26705-x. 34732730 PMC8566515

[ref50] Song N.; Zhu H.; Jin S.; Zhan W.; Lian T. Poisson-Distributed Electron-Transfer Dynamics from Single Quantum Dots to C60 Molecules. ACS Nano 2011, 5 (1), 613–621. 10.1021/nn1028828. 21190376

[ref51] Song N.; Zhu H.; Jin S.; Lian T. Hole Transfer from Single Quantum Dots. ACS Nano 2011, 5 (11), 8750–8759. 10.1021/nn202713x. 21962001

[ref52] Morris-Cohen A. J.; Frederick M. T.; Cass L. C.; Weiss E. A. Simultaneous Determination of the Adsorption Constant and the Photoinduced Electron Transfer Rate for a Cds Quantum Dot-Viologen Complex. J. Am. Chem. Soc. 2011, 133 (26), 10146–10154. 10.1021/ja2010237. 21618976

[ref53] Kern S. J.; Sahu K.; Berg M. A. Heterogeneity of the Electron-Trapping Kinetics in CdSe Nanoparticles. Nano Lett. 2011, 11 (8), 3493–3498. 10.1021/nl202086b. 21780773

[ref54] Hu K.; Blair A. D.; Piechota E. J.; Schauer P. A.; Sampaio R. N.; Parlane F. G. L.; Meyer G. J.; Berlinguette C. P. Kinetic pathway for interfacial electron transfer from a semiconductor to a molecule. Nat. Chem. 2016, 8 (9), 853–859. 10.1038/nchem.2549. 27554412

[ref55] Ding T. X.; Olshansky J. H.; Leone S. R.; Alivisatos A. P. Efficiency of Hole Transfer from Photoexcited Quantum Dots to Covalently Linked Molecular Species. J. Am. Chem. Soc. 2015, 137 (5), 2021–2029. 10.1021/ja512278a. 25591013

[ref56] Padgaonkar S.; Eckdahl C. T.; Sowa J. K.; López-Arteaga R.; Westmoreland D. E.; Woods E. F.; Irgen-Gioro S.; Nagasing B.; Seideman T.; Hersam M. C.; Kalow J. A.; Weiss E. A. Light-Triggered Switching of Quantum Dot Photoluminescence through Excited-State Electron Transfer to Surface-Bound Photochromic Molecules. Nano Lett. 2021, 21 (1), 854–860. 10.1021/acs.nanolett.0c04611. 33395307

[ref57] Pitre S. P.; McTiernan C. D.; Scaiano J. C. Understanding the Kinetics and Spectroscopy of Photoredox Catalysis and Transition-Metal-Free Alternatives. Acc. Chem. Res. 2016, 49 (6), 1320–30. 10.1021/acs.accounts.6b00012. 27023767

[ref58] Kandoth N.; Pérez Hernández J.; Palomares E.; Lloret-Fillol J. Mechanisms of photoredox catalysts: the role of optical spectroscopy. Sustainable Energy & Fuels 2021, 5 (3), 638–665. 10.1039/D0SE01454K.

[ref59] Laferrière M.; Galian R. E.; Maurel V.; Scaiano J. C. Non-linear effects in the quenching of fluorescent quantum dots by nitroxyl free radicals. Chem. Commun. 2006, (3), 257–259. 10.1039/B511515A. 16391726

[ref60] Sarkar S.; Ravi V. K.; Banerjee S.; Yettapu G. R.; Markad G. B.; Nag A.; Mandal P. Terahertz Spectroscopic Probe of Hot Electron and Hole Transfer from Colloidal CsPbBr3 Perovskite Nanocrystals. Nano Lett. 2017, 17 (9), 5402–5407. 10.1021/acs.nanolett.7b02003. 28831807

[ref61] Goodman A. J.; Dahod N. S.; Tisdale W. A. Ultrafast Charge Transfer at a Quantum Dot/2D Materials Interface Probed by Second Harmonic Generation. J. Phys. Chem. Lett. 2018, 9 (15), 4227–4232. 10.1021/acs.jpclett.8b01606. 29995420

[ref62] Wang C.; Li Y.; Xiong W. Extracting molecular responses from ultrafast charge dynamics at material interfaces. J. Mater. Chem. C 2020, 8 (35), 12062–12067. 10.1039/D0TC01819H.

[ref63] Mu C.; Lv C.; Meng X.; Sun J.; Tong Z.; Huang K. In Situ Characterization Techniques Applied in Photocatalysis: A Review. Advanced Materials Interfaces 2023, 10 (3), 2201842 10.1002/admi.202201842.

[ref64] Zhang Y.; Hu H.; Huang X.; Bi Y. Photo-controlled bond changes on Pt/TiO2 for promoting overall water splitting and restraining hydrogen-oxygen recombination. J. Mater. Chem. A 2019, 7 (11), 5938–5942. 10.1039/C8TA11595H.

[ref65] Zhang L.; Zhang Y.; Huang X.; Bi Y. Reversing electron transfer in a covalent triazine framework for efficient photocatalytic hydrogen evolution. Chem. Sci. 2022, 13 (27), 8074–8079. 10.1039/D2SC02638D. 35919433 PMC9278156

[ref66] Wang Y.; Ma Y.; Li X.-B.; Gao L.; Gao X.-Y.; Wei X.-Z.; Zhang L.-P.; Tung C.-H.; Qiao L.; Wu L.-Z. Unveiling Catalytic Sites in a Typical Hydrogen Photogeneration System Consisting of Semiconductor Quantum Dots and 3d-Metal Ions. J. Am. Chem. Soc. 2020, 142 (10), 4680–4689. 10.1021/jacs.9b11768. 32066243

[ref67] Chen L. X. Taking Snapshots of Photoexcited Molecules in Disordered Media by Using Pulsed Synchrotron X-rays. Angew. Chem., Int. Ed. 2004, 43 (22), 2886–2905. 10.1002/anie.200300596. 15170299

[ref68] Chen L. X.; Zhang X. Photochemical Processes Revealed by X-ray Transient Absorption Spectroscopy. J. Phys. Chem. Lett. 2013, 4 (22), 4000–4013. 10.1021/jz401750g.

[ref69] Li Z.-J.; Zhan F.; Xiao H.; Zhang X.; Kong Q.-Y.; Fan X.-B.; Liu W.-Q.; Huang M.-Y.; Huang C.; Gao Y.-J.; Li X.-B.; Meng Q.-Y.; Feng K.; Chen B.; Tung C.-H.; Zhao H.-F.; Tao Y.; Wu L.-Z. Tracking Co(I) Intermediate in Operando in Photocatalytic Hydrogen Evolution by X-ray Transient Absorption Spectroscopy and DFT Calculation. J. Phys. Chem. Lett. 2016, 7 (24), 5253–5258. 10.1021/acs.jpclett.6b02479. 27973864

[ref70] Chen R.; Fan F.; Dittrich T.; Li C. Imaging photogenerated charge carriers on surfaces and interfaces of photocatalysts with surface photovoltage microscopy. Chem. Soc. Rev. 2018, 47 (22), 8238–8262. 10.1039/C8CS00320C. 30059114

[ref71] Chen R.; Ren Z.; Liang Y.; Zhang G.; Dittrich T.; Liu R.; Liu Y.; Zhao Y.; Pang S.; An H.; Ni C.; Zhou P.; Han K.; Fan F.; Li C. Spatiotemporal imaging of charge transfer in photocatalyst particles. Nature 2022, 610 (7931), 296–301. 10.1038/s41586-022-05183-1. 36224420

[ref72] Homer M. K.; Kuo D.-Y.; Dou F. Y.; Cossairt B. M. Photoinduced Charge Transfer from Quantum Dots Measured by Cyclic Voltammetry. J. Am. Chem. Soc. 2022, 144 (31), 14226–14234. 10.1021/jacs.2c04991. 35897128

[ref73] Nyakuchena J.; Zhang X.; Huang J. Synchrotron based transient x-ray absorption spectroscopy for emerging solid-state energy materials. Chem. Phys. Rev. 2023, 4 (2), 021303 10.1063/5.0133227.

[ref74] Mahjour B.; Shen Y.; Cernak T. Ultrahigh-Throughput Experimentation for Information-Rich Chemical Synthesis. Acc. Chem. Res. 2021, 54 (10), 2337–2346. 10.1021/acs.accounts.1c00119. 33891404

[ref75] Mai H.; Le T. C.; Chen D.; Winkler D. A.; Caruso R. A. Machine Learning for Electrocatalyst and Photocatalyst Design and Discovery. Chem. Rev. 2022, 122 (16), 13478–13515. 10.1021/acs.chemrev.2c00061. 35862246

[ref76] Huang C.; Qiao J.; Ci R.-N.; Wang X.-Z.; Wang Y.; Wang J.-H.; Chen B.; Tung C.-H.; Wu L.-Z. Quantum dots enable direct alkylation and arylation of allylic C(sp3)-H bonds with hydrogen evolution by solar energy. Chem. 2021, 7 (5), 1244–1257. 10.1016/j.chempr.2021.01.019.

[ref77] Vinu R.; Madras G. Kinetics of Simultaneous Photocatalytic Degradation of Phenolic Compounds and Reduction of Metal Ions with Nano-TiO2. Environ. Sci. Technol. 2008, 42 (3), 913–919. 10.1021/es0720457. 18323122

[ref78] Tanaka D.; Oaki Y.; Imai H. Enhanced photocatalytic activity of quantum-confined tungsten trioxide nanoparticles in mesoporous silica. Chem. Commun. 2010, 46 (29), 5286–5288. 10.1039/c0cc00540a. 20563333

[ref79] Li X.-B.; Li Z.-J.; Gao Y.-J.; Meng Q.-Y.; Yu S.; Weiss R. G.; Tung C.-H.; Wu L.-Z. Mechanistic Insights into the Interface-Directed Transformation of Thiols into Disulfides and Molecular Hydrogen by Visible-Light Irradiation of Quantum Dots. Angew. Chem., Int. Ed. 2014, 53 (8), 2085–2089. 10.1002/anie.201310249. 24470069

[ref80] Gao Y.-J.; Li X.-B.; Wang X.-Z.; Zhao N.-J.; Zhao Y.; Wang Y.; Xin Z.-K.; Zhang J.-P.; Zhang T.; Tung C.-H.; Wu L.-Z. Site- and Spatial-Selective Integration of Non-noble Metal Ions into Quantum Dots for Robust Hydrogen Photogeneration. Matter 2020, 3 (2), 571–585. 10.1016/j.matt.2020.06.022.

[ref81] Pal A.; Ghosh I.; Sapra S.; König B. Quantum Dots in Visible-Light Photoredox Catalysis: Reductive Dehalogenations and C-H Arylation Reactions Using Aryl Bromides. Chem. Mater. 2017, 29 (12), 5225–5231. 10.1021/acs.chemmater.7b01109.

[ref82] Huang C.; Li X.-B.; Tung C.-H.; Wu L.-Z. Photocatalysis with Quantum Dots and Visible Light for Effective Organic Synthesis. Chem.—Eur. J. 2018, 24 (45), 11530–11534. 10.1002/chem.201800391. 29575190

[ref83] Huang C.; Ci R.-N.; Qiao J.; Wang X.-Z.; Feng K.; Chen B.; Tung C.-H.; Wu L.-Z. Direct Allylic C(sp3)-H and Vinylic C(sp2)-H Thiolation with Hydrogen Evolution by Quantum Dots and Visible Light. Angew. Chem., Int. Ed. 2021, 60 (21), 11779–11783. 10.1002/anie.202101947. 33660909

[ref84] Ci R.-N.; Huang C.; Zhao L.-M.; Qiao J.; Chen B.; Feng K.; Tung C.-H.; Wu L.-Z. General and Efficient C-P Bond Formation by Quantum Dots and Visible Light. CCS Chemistry 2022, 4 (9), 2946–2952. 10.31635/ccschem.021.202101615.

[ref85] Gan Q.-C.; Qiao J.; Zhou C.; Ci R.-N.; Guo J.-D.; Chen B.; Tung C.-H.; Wu L.-Z. Direct N-H Activation to Generate Nitrogen Radical for Arylamine Synthesis via Quantum Dots Photocatalysis. Angew. Chem., Int. Ed. 2023, 62 (17), e202218391 10.1002/anie.202218391. 36808675

[ref86] Meng Q.-Y.; Zhong J.-J.; Liu Q.; Gao X.-W.; Zhang H.-H.; Lei T.; Li Z.-J.; Feng K.; Chen B.; Tung C.-H.; Wu L.-Z. A Cascade Cross-Coupling Hydrogen Evolution Reaction by Visible Light Catalysis. J. Am. Chem. Soc. 2013, 135 (51), 19052–19055. 10.1021/ja408486v. 24160446

[ref87] Chen B.; Wu L.-Z.; Tung C.-H. Photocatalytic Activation of Less Reactive Bonds and Their Functionalization via Hydrogen-Evolution Cross-Couplings. Acc. Chem. Res. 2018, 51 (10), 2512–2523. 10.1021/acs.accounts.8b00267. 30280898

[ref88] Qiao J.; Song Z.-Q.; Huang C.; Ci R.-N.; Liu Z.; Chen B.; Tung C.-H.; Wu L.-Z. Direct, Site-Selective and Redox-Neutral α-C-H Bond Functionalization of Tetrahydrofurans via Quantum Dots Photocatalysis. Angew. Chem., Int. Ed. 2021, 60 (52), 27201–27205. 10.1002/anie.202109849. 34536248

[ref89] Qiao J.; Ci R.-N.; Gan Q.-C.; Huang C.; Liu Z.; Hu H.-L.; Ye C.; Chen B.; Tung C.-H.; Wu L.-Z. Amine-Free, Directing-Group-Free and Redox-Neutral α-Alkylation of Saturated Cyclic Ketones. Angew. Chem., Int. Ed. 2023, 62 (29), e202305679 10.1002/anie.202305679. 37218528

[ref90] Arcudi F.; Đorđević L.; Nagasing B.; Stupp S. I.; Weiss E. A. Quantum Dot-Sensitized Photoreduction of CO2 in Water with Turnover Number > 80,000. J. Am. Chem. Soc. 2021, 143 (43), 18131–18138. 10.1021/jacs.1c06961. 34664969

[ref91] Lian S.; Kodaimati M. S.; Weiss E. A. Photocatalytically Active Superstructures of Quantum Dots and Iron Porphyrins for Reduction of CO2 to CO in Water. ACS Nano 2018, 12 (1), 568–575. 10.1021/acsnano.7b07377. 29298382

[ref92] Nie C.; Lin X.; Zhao G.; Wu K. Low-Toxicity ZnSe/ZnS Quantum Dots as Potent Photoreductants and Triplet Sensitizers for Organic Transformations. Angew. Chem., Int. Ed. 2022, 61 (49), e202213065 10.1002/anie.202213065. 36250269

[ref93] Weinberg D. J.; He C.; Weiss E. A. Control of the Redox Activity of Quantum Dots through Introduction of Fluoroalkanethiolates into Their Ligand Shells. J. Am. Chem. Soc. 2016, 138 (7), 2319–2326. 10.1021/jacs.5b13077. 26820492

[ref94] McClelland K. P.; Weiss E. A. Selective Photocatalytic Oxidation of Benzyl Alcohol to Benzaldehyde or C-C Coupled Products by Visible-Light-Absorbing Quantum Dots. ACS Appl. Energy Mater. 2019, 2 (1), 92–96. 10.1021/acsaem.8b01652.

[ref95] Widness J. K.; Enny D. G.; McFarlane-Connelly K. S.; Miedenbauer M. T.; Krauss T. D.; Weix D. J. CdS Quantum Dots as Potent Photoreductants for Organic Chemistry Enabled by Auger Processes. J. Am. Chem. Soc. 2022, 144 (27), 12229–12246. 10.1021/jacs.2c03235. 35772053 PMC9306379

[ref96] Lian S.; Christensen J. A.; Kodaimati M. S.; Rogers C. R.; Wasielewski M. R.; Weiss E. A. Oxidation of a Molecule by the Biexcitonic State of a CdS Quantum Dot. J. Phys. Chem. C 2019, 123 (10), 5923–5930. 10.1021/acs.jpcc.9b00210.

[ref97] Perez K. A.; Rogers C. R.; Weiss E. A. Quantum Dot-Catalyzed Photoreductive Removal of Sulfonyl-Based Protecting Groups. Angew. Chem., Int. Ed. 2020, 59 (33), 14091–14095. 10.1002/anie.202005074. PMC7480491 32396699

[ref98] Jensen S. C.; Bettis Homan S.; Weiss E. A. Photocatalytic Conversion of Nitrobenzene to Aniline through Sequential Proton-Coupled One-Electron Transfers from a Cadmium Sulfide Quantum Dot. J. Am. Chem. Soc. 2016, 138 (5), 1591–1600. 10.1021/jacs.5b11353. 26784531

[ref99] Mouat J. M.; Widness J. K.; Enny D. G.; Meidenbauer M. T.; Awan F.; Krauss T. D.; Weix D. J. CdS Quantum Dots for Metallaphotoredox-Enabled Cross-Electrophile Coupling of Aryl Halides with Alkyl Halides. ACS Catal. 2023, 13, 9018–9024. 10.1021/acscatal.3c01984. 38283073 PMC10812861

[ref100] Yuan Y.; Zhu H.; Hills-Kimball K.; Cai T.; Shi W.; Wei Z.; Yang H.; Candler Y.; Wang P.; He J.; Chen O. Stereoselective C-C Oxidative Coupling Reactions Photocatalyzed by Zwitterionic Ligand Capped CsPbBr3 Perovskite Quantum Dots. Angew. Chem., Int. Ed. 2020, 59 (50), 22563–22569. 10.1002/anie.202007520. 32852841

[ref101] Wang X.; Li C. Interfacial charge transfer in semiconductor-molecular photocatalyst systems for proton reduction. Journal of Photochemistry and Photobiology C: Photochemistry Reviews 2017, 33, 165–179. 10.1016/j.jphotochemrev.2017.10.003.

[ref102] Li H.; Sun C.; Ali M.; Zhou F.; Zhang X.; MacFarlane D. R. Sulfated Carbon Quantum Dots as Efficient Visible-Light Switchable Acid Catalysts for Room-Temperature Ring-Opening Reactions. Angew. Chem., Int. Ed. 2015, 54 (29), 8420–8424. 10.1002/anie.201501698. 26032183

